# Investigation of a novel *TBC1D24* variation causing autosomal dominant non-syndromic hearing loss

**DOI:** 10.1038/s41598-024-55435-5

**Published:** 2024-02-27

**Authors:** Peiliang Lei, Qingwen Zhu, Wenrong Dong

**Affiliations:** 1 Department of Otolaryngology Head & Neck Surgery, The Second Hospital of Hebei Medical University, Heping West Road No. 215, Shijiazhuang, 050000 Hebei China; 2 Department of Otolaryngology Head & Neck Surgery, The Third Hospital of Shijiazhuang, Tiyu South Street No.15, Shijiazhuang, 050011 Hebei China

**Keywords:** *TBC1D24*, Hearing loss, Gene mutation, Hereditary deafness, Genetics research, Clinical genetics

## Abstract

Hearing loss is considered one of the most common sensory neurological defects, with approximately 60% of cases attributed to genetic factors. Human pathogenic variants in the *TBC1D24* gene are associated with various clinical phenotypes, including dominant nonsyndromic hearing loss DFNA65, characterized by progressive hearing loss after the development of language. This study provides an in-depth analysis of the causative gene and mutations in a family with hereditary deafness. We recruited a three-generation family with autosomal dominant nonsyndromic hearing loss (ADNSHL) and conducted detailed medical histories and relevant examinations. Next-generation sequencing (NGS) was used to identify genetic variants in the proband, which were then validated using Sanger sequencing. Multiple computational software tools were employed to predict the impact of the variant on the function and structure of the *TBC1D24* protein. A series of bioinformatics tools were applied to determine the conservation characteristics of the sequence, establish a three-dimensional structural model, and investigate changes in molecular dynamics. A detailed genotype and phenotype analysis were carried out. The family exhibited autosomal dominant, progressive, postlingual, and nonsyndromic sensorineural hearing loss. A novel heterozygous variant, c.1459C>T (p.His487Tyr), in the *TBC1D24* gene was identified and confirmed to be associated with the hearing loss phenotype in this family. Conservation analysis revealed high conservation of the amino acid affected by this variant across different species. The mutant protein showed alterations in thermodynamic stability, elasticity, and conformational dynamics. Molecular dynamics simulations indicated changes in RMSD, RMSF, Rg, and SASA of the mutant structure. We computed the onset age of non-syndromic hearing loss associated with mutations in the *TBC1D24* gene and identified variations in the hearing progression time and annual threshold deterioration across different frequencies. The identification of a new variant associated with rare autosomal dominant nonsyndromic hereditary hearing loss in this family broadens the range of mutations in the *TBC1D24* gene. This variant has the potential to influence the interaction between the TLDc domain and TBC domain, thereby affecting the protein’s biological function.

## Introduction

Hearing loss is a prevalent and diverse sensory impairment. According to the World Health Organization, more than 5% of the global population, which amounts to 466 million people, experience varying degrees of hearing loss^1^. Currently, researchers have identified a total of 124 genes associated with hereditary non-syndromic hearing loss. These genes include 51 for autosomal dominant hearing loss, 78 for autosomal recessive hearing loss, and 5 for X-linked inheritance^2^. Autosomal dominant non-syndromic hearing loss (ADNSHL) constitutes approximately 20% of cases of non-syndromic hereditary hearing loss^3^. Hereditary hearing loss demonstrates significant genetic heterogeneity, with multiple genes implicated in both autosomal recessive and autosomal dominant forms of the condition. The clinical presentations of hereditary hearing loss are diverse, contributing to its varied characteristics^4^. While certain hearing loss genes can cause isolated deafness, they can also give rise to phenotypes that involve additional symptoms alongside hearing loss. The *TBC1D24* gene serves as a notable example of this phenomenon.

*TBC1D24* (MIM #613577) is the 24th member of the TBC1 structural domain gene family, situated on chromosome 16p13.3. It consists of eight exons, with the longest mRNA encoding a polypeptide comprising 559 amino acids. Two conserved structural domains, TBC and TLDc, are encoded by amino acids 47 to 262 and 368 to 554, respectively^5^. Over 40 distinct TBC proteins (TBC domain-containing proteins) have been identified in humans. This protein family falls under the category of Rab-GTPase-activating proteins (Rab-GAPs), which facilitate the hydrolysis of GTP from specific Rab-GTPases, thereby regulating their activation state^6^. By modulating the activity of Rab-GTPases, Rab-GAPs play a role in governing numerous processes involving plasma membrane transport and vesicle sorting.

Multiple studies^7–14^ have documented both dominant and recessive non-syndromic deafness, as well as syndromic deafness, resulting from mutations in the *TBC1D24* gene. These findings indicate the gene’s crucial role in the auditory system. In this study, we identify a novel heterozygous variation, c.1459C>T (p.His487Tyr), in a Chinese family. These results expand the range of pathogenic variants associated with the *TBC1D24* gene and further support the diagnosis and characterization of hereditary deafness caused by such variants.

## Methods

### Ethics approval

All participants in the study provided informed consent to enroll in the research after following the ten principles of the Declaration of Helsinki. We confirmed that all experiments were performed in accordance with relevant guidelines and regulations. All procedures were approved by the Ethics Reviewing Committee of the Second Hospital of Hebei Medical University. Written informed consent was obtained from all subjects or their parents for their participation in the study.

### Pedigree and clinical evaluation

**Figure 1:**
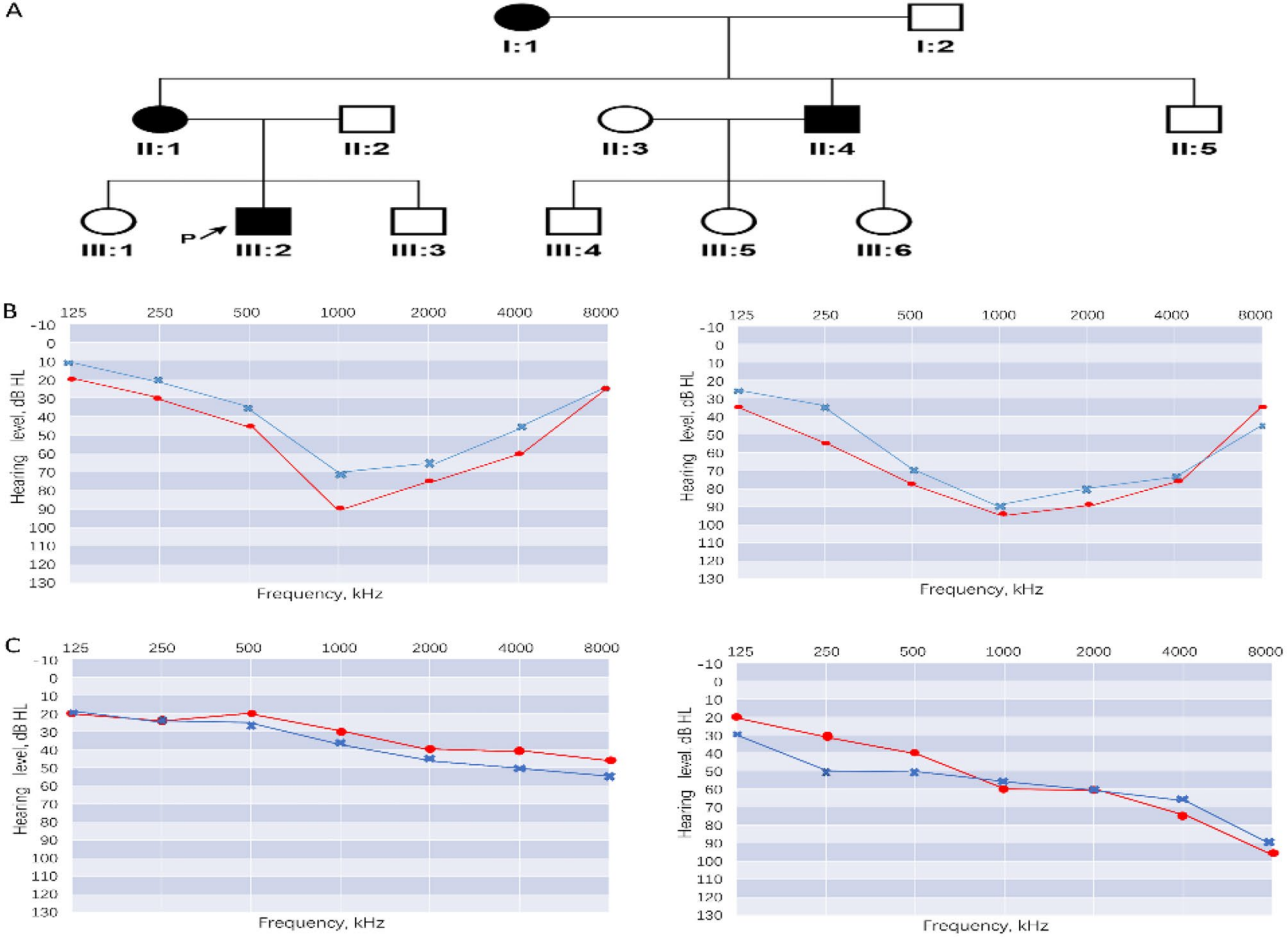
(**A**) Pedigreed diagrams of the family with autosomal dominant hearing loss (symbols with line, deceased). The proband is indicated by an arrow. The affected subjects are denoted in black. (**B**) Audiograms of the proband at the ages of 14 and 20. (**C**) Audiograms of other affected subjects (red: right ear; blue: left ear).

We collected a three-generation family affected by hearing loss from Handan City in Hebei Province (Fig. 1A). A questionnaire was administered to gather detailed medical histories from family members, including information on age at onset and triggers, subjective degree of hearing loss, presence of tinnitus and vertigo, progression of hearing loss, history of head trauma, medication with aminoglycosides, noise exposure, pathological changes in the ear, and other relevant clinical presentations.

All subjects underwent an otolaryngological clinical examination, which included otoscopy, physical examination, pure tone audiometry (frequencies ranging from 0.125 to 8 kHz), acoustic conduction resistance, and computed tomography of the temporal bone. The hearing curve was plotted to determine the degree and type of hearing loss. The diagnosis of sensorineural hearing loss was based on the criteria established by the World Health Organization.

### Genetic analysis and validation

Genomic DNA was extracted from peripheral blood samples of family members using a blood DNA extraction kit (TIANGEN, Beijing, China). The Standard Library Building kit (MyGenostics GenCap Enrichment Technologies) was used according to the manufacturer’s instructions. This involved ultrasonic fragmentation of DNA fragments, DNA library construction, hybridization capture, and capture library amplification and purification. The GenCap deafness capture kit (MyGenostics, Beijing, China) was used to capture the qualified library, which covers 415 genes including prevalent deafness genes such as GJB2, SLC26A4, and mtDNA12SrRNA. We selected exons and their flanking 100 bp for capture, and NGS sequencing was performed on an Illumina HiSeq2000 (Illumina, San Diego, CA, USA).

Raw data generated by NGS were filtered to obtain high-quality clean reads and aligned to the NCBI Human Reference Genome (hg19/GRCh37) using the Burrows Wheeler Aligner (BWA). Candidate pathogenic variants were filtered with a minimum allele frequency threshold of ≤ 0.001 for dominant inheritance. Sanger sequencing was used to validate the co-segregation of the variant with the phenotype. Computational programs such as SIFT (http://sift.jcvi.org/), GERP++^15^, Mutation Taster (http://www.mutationtaster.org/), Mutpred^16^, Revel^17^, CADD^18^, DANN^19^ and were employed to determine the pathogenicity of candidate variants. The sequencing analysis described above was conducted at Beijing MyGenostics Company. Data interpretation followed the guidelines provided by the American College of Medical Genetics and Genomics (ACMG).

### Structure modeling and analysis

To analyze the conservation of the mutation site, we extracted the wild-type *TBC1D24* sequence from the NCBI protein database. SeqLogo generated a sequence logo representation to illustrate the conservation pattern. We utilized the VMD plugin, VMD-SS, to analyze secondary structure information and the composition of each secondary structure element. Ramachandran plots were used to visualize the distribution of energetically allowed regions for amino acid residues in the peptide backbone. The MolProbity online tool (http://molprobity.biochem.duke.edu/) was employed to assess the stereochemical quality of the protein structure.

The SWISS-MODEL automated homology modeling program was used to model the wild-type and mutant structures of the *TBC1D24* gene in three dimensions. The resulting models were visualized using VMD. Qualitative electrostatic representations of the wild-type and mutant structures were generated using PyMOL to highlight their distinguishing features. Additionally, the local structural characteristics of the wild-type and mutant proteins were assessed using the DynaMut^20^ online platform to predict thermodynamic stability changes before and after the mutation. The findings were rendered and visualized using PyMOL to facilitate a comprehensive understanding of the analysis outcomes.

### Molecular dynamics simulation

To investigate the potential functional impact of this missense mutation, we conducted molecular dynamics simulations (MDS) using Gromacs 2018.1 on the Oracle Cloud platform. The energetically minimized structures obtained from SWISS-MODEL served as the starting structures, and the charmm27 force field within the Gromacs software package was employed for the simulation.

A cubic box with a side length of 10 nm was set up around the protein molecule, and SPC water molecules were used to solvate the box. Counter ions were added for neutralization, and initial energy minimization was performed using the steepest descent method. System equilibration was then carried out in both the isothermal-isobaric ensemble (NPT) and the canonical ensemble (NVT), with each step consisting of 50,000 steps to ensure stable volume, pressure, and temperature.

Finally, a 100 ns MD simulation was conducted to analyze parameters such as conformational changes and structural stability of the protein throughout the simulation. This allowed us to assess the potential effects of the mutation on protein function. Comparative analyses of structural deviations, including RMSD, RMSF, Rg, and SASA, were performed using tools such as g_rms, g_rmsf, g_Rg, and g_sasa. All figures were generated using the XMGRACE software.

### Audiometric analyses

We conducted a literature search and meta-analysis using the PubMed database to obtain the hearing data of families with *TBC1D24* mutation-related non-syndromic hearing loss. We selected the patient records that contained age information and pure tone audiograms (bilaterally symmetric). First, we calculated the mean values of bilateral air conduction thresholds at 500, 1000, 2000, and 4000 Hz, and then performed linear and non-linear regression analyses to estimate the onset age. We defined the onset age as the number obtained from the regression equation when the hearing threshold exceeded 25 dB. Next, we generated the audiograms of age versus hearing threshold for three frequencies: low (250 Hz, 500 Hz), mid (1000 Hz, 2000 Hz), and high (4000 Hz, 8000 Hz), and performed regression analyses. We defined the hearing progression time (HPT) as the time required for the hearing threshold to drop from 25 to 60 dB and calculated the differences among the three frequencies using the fitted equations. Finally, we separated the patients’ hearing data into two groups, TBC and TLDc, according to the localization of the domains of the mutations, and compared the differences in annual threshold deterioration (ATD) between the two groups. The analyses were performed using SPSS software (version 23).

## Results

### Clinical manifestations

Figure 1A shows a pedigree with three generations, including four individuals affected by hearing impairment. The audiograms of the index case at ages 14 and 20 are depicted in Fig. 1B, while Fig. 1C shows the audiograms of two additional affected family members. None of the patients exhibited vestibular dysfunction or had a significant history of neurological disorders. All affected individuals presented with bilateral progressive sensorineural hearing loss. Apart from the proband, the other three members experienced a gradual onset of hearing loss in adulthood. It should be noted that these individuals underwent testing several years after the onset of symptoms, making it difficult to determine the specific type of hearing loss during the initial stages of impairment.

### Identification and validation of the mutation

The targeted regions had an average sequencing depth of 917.17-fold. The coverage for the targeted exons was 98.85% at 10× and 98.36% at 20×. In the *TBC1D24* gene, we discovered a novel heterozygous missense mutation c.1459C>T (p.His487Tyr). This mutation was carried by the proband and his mother, but not by the father. We validated the variant using Sanger sequencing, and it co-segregated with the phenotype. Individuals I:1, II:1, II:4, and III:2, who carried the variant at this site, exhibited clinical phenotypes, while unaffected members did not have this mutation.

To analyze the identified variant, we consulted the NCBI dbSNP database and the 1000 Genomes Project database. Additionally, we searched the Human Gene Mutation Database (HGMD, http://www.hgmd.org/) and PubMed (http://www.ncbi.nlm.nih.gov/pubmed/) databases, which confirmed that this specific base change had not been previously reported. Functional prediction tools such as SIFT, GERP, Mutation Taster Mutpred, Revel, CADD, and DANN predicted the mutation as “Uncertain”, “Uncertain”, “Deleterious”, “Pathogenic supporting”, “Uncertain”, “Deleterious”, and “Deleterious”, respectively (Table 1). Based on the ACMG classification, this variant was categorized as likely pathogenic (PM2, PP1_strong).

**Table 1:** Pathogenicity assessment in silico of TBC1D24: c.1459C>T (p.His487Tyr).

	SIFT	GERP	Mutation Taster	Mutpred	Revel	CADD	DANN
Scores	0.008	5.49	1	0.679	0.31	23.0	1
Pathogenicity	Uncertain	Uncertain	Deleterious	Pathogenic supporting	Uncertain	Deleterious	Deleterious

### Analysis of the static structure

**Figure 2:**
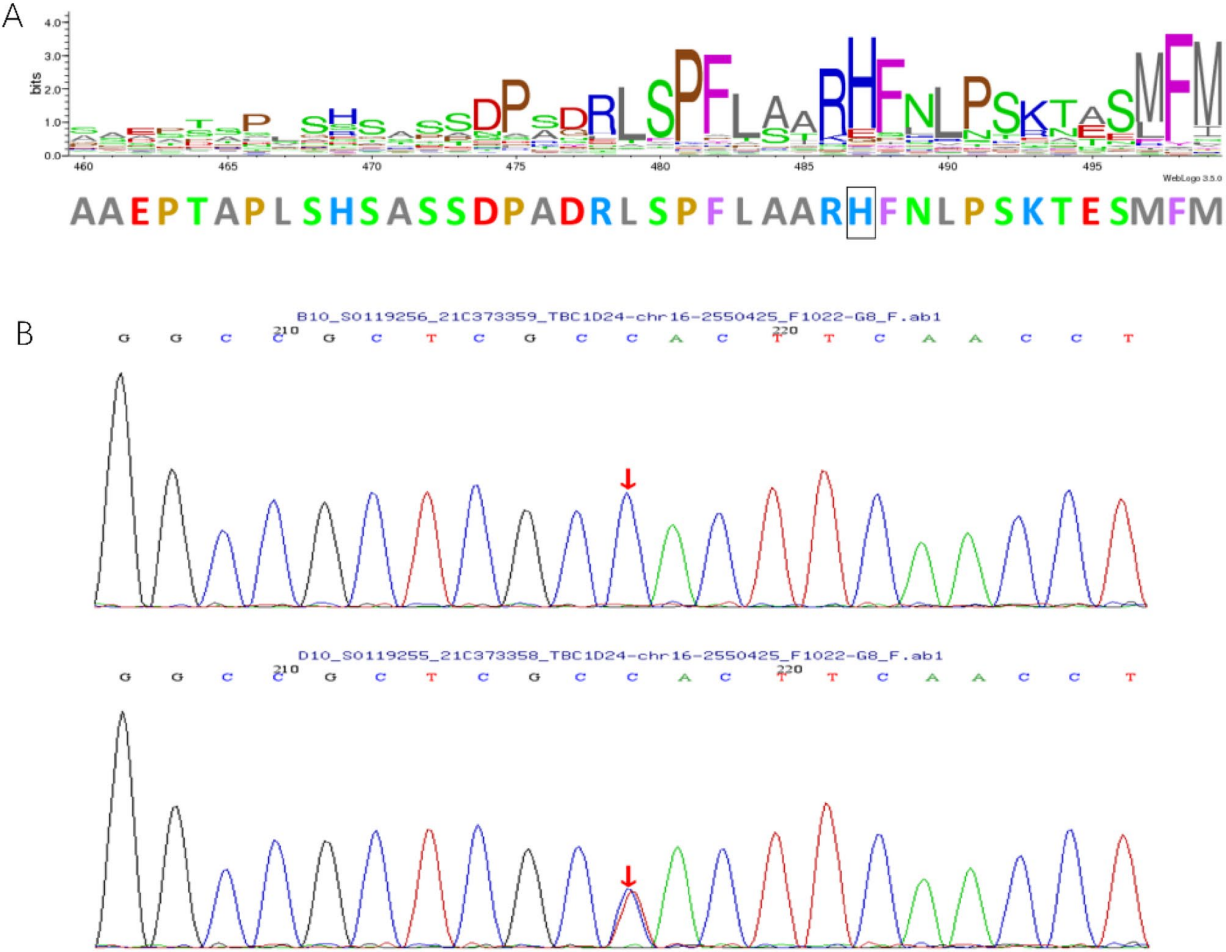
(**A**) Protein alignment shows the conservation of the H487 residue of the *TBC1D24*. (**B**) Mutation detection shows the heterozygote c.1459C>T mutation in the proband.

The identified variant is a missense mutation within the TLDc functional domain, resulting in the substitution of histidine with tyrosine at amino acid position 487 of the *TBC1D24* protein. Evolutionary conservation analysis showed that the histidine residue at position 487 is conserved across multiple vertebrate species (Fig. 2).

**Figure 3:**
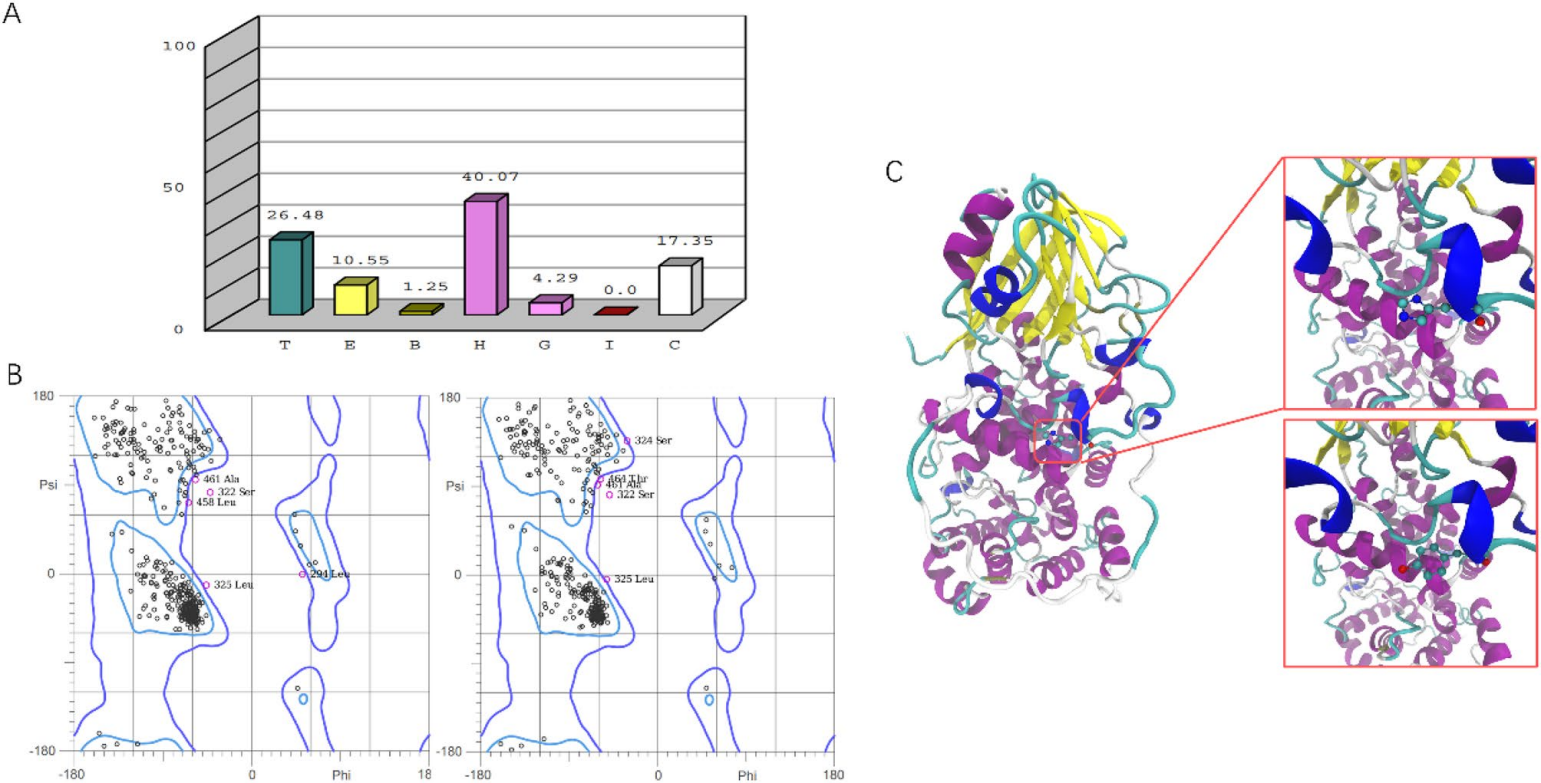
(**A**) The secondary structures of the *TBC1D24* protein include turn (T), fold (E), β-sheet (B), α-helix (H), 3-turn helix (**G**), 5-turn helix (I), and random coil (C). (**B**) Comparison of the Ramachandran plots of the *TBC1D24* protein. (**C**) Predicted structures depict the changes of mutant protein with the amino acid change H487Y.

Figure 3A shows the composition ratio of various secondary structures of the *TBC1D24* protein. Ramachandran plot analysis (Fig. 3B) comparing the wild-type and mutant forms indicated no significant impact. 98.9% and 99.1% of the total residues fell within the allowed regions, respectively. Homology modeling of the wild-type and mutant forms enabled an assessment of the structural consequences of this mutation (Fig. 3C).

**Figure 4:**
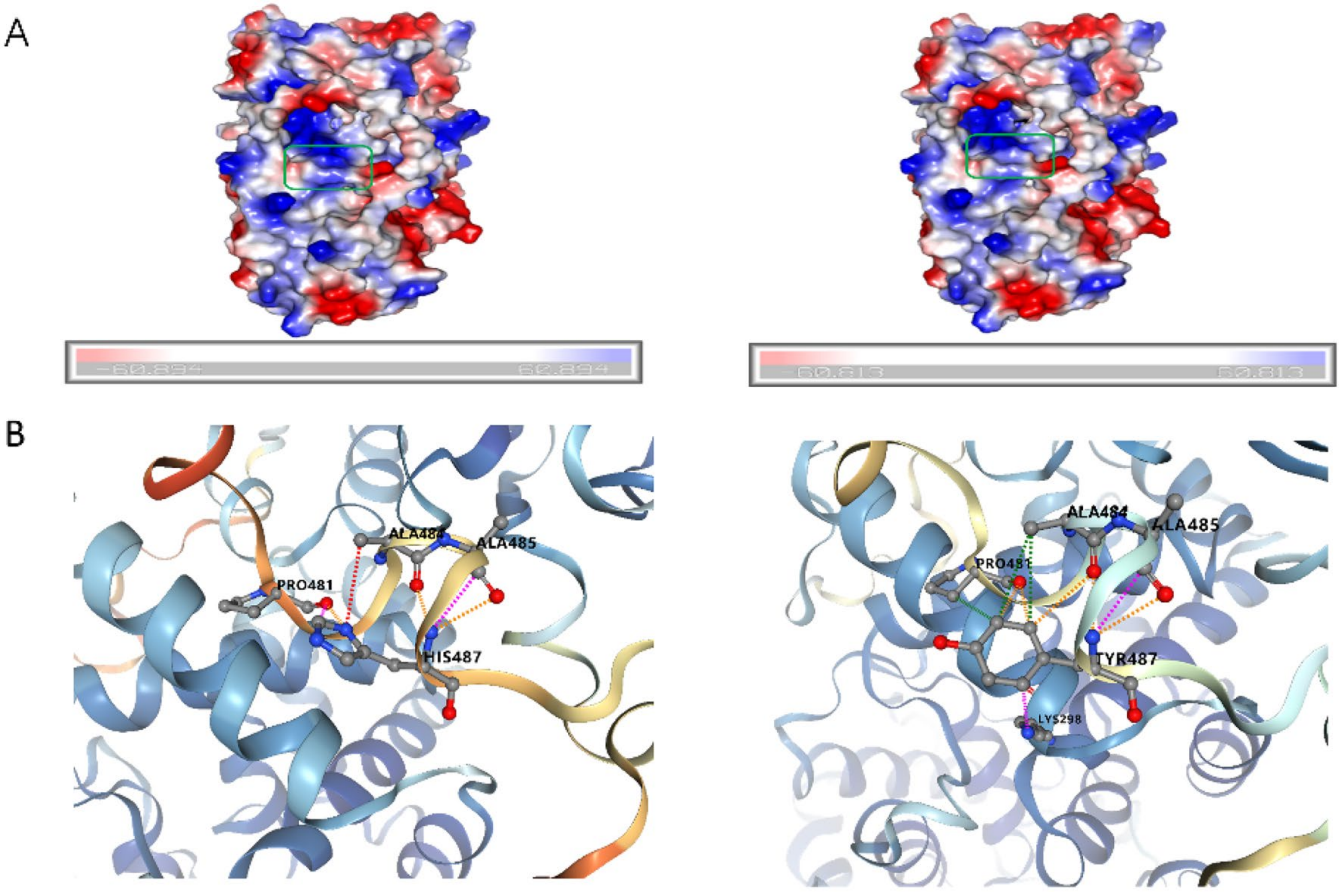
(**A**) The qualitative electrostatic representation of the *TBC1D24* protein and the mutant H487Y protein generated by PyMOL. Protein contact potentials can be represented by displaying virtual (false) red/blue charged smooth surfaces. (**B**) The changes between amino acids before and after mutation.

Protein contact potential analysis supported a decrease in electrostatic potential at the mutation site (Fig. 4A). Figure 4B presents the predicted local interactions of the amino acid residues before and after the mutation using DynaMut, including hydrogen bonds and disulfide bonds. This mutation induces changes in the interactions between the residue at position 487 and neighboring amino acids, as well as interactions with the amino acid at position 298 within the TBC domain.

### Analysis of molecular dynamics

**Figure 5:**
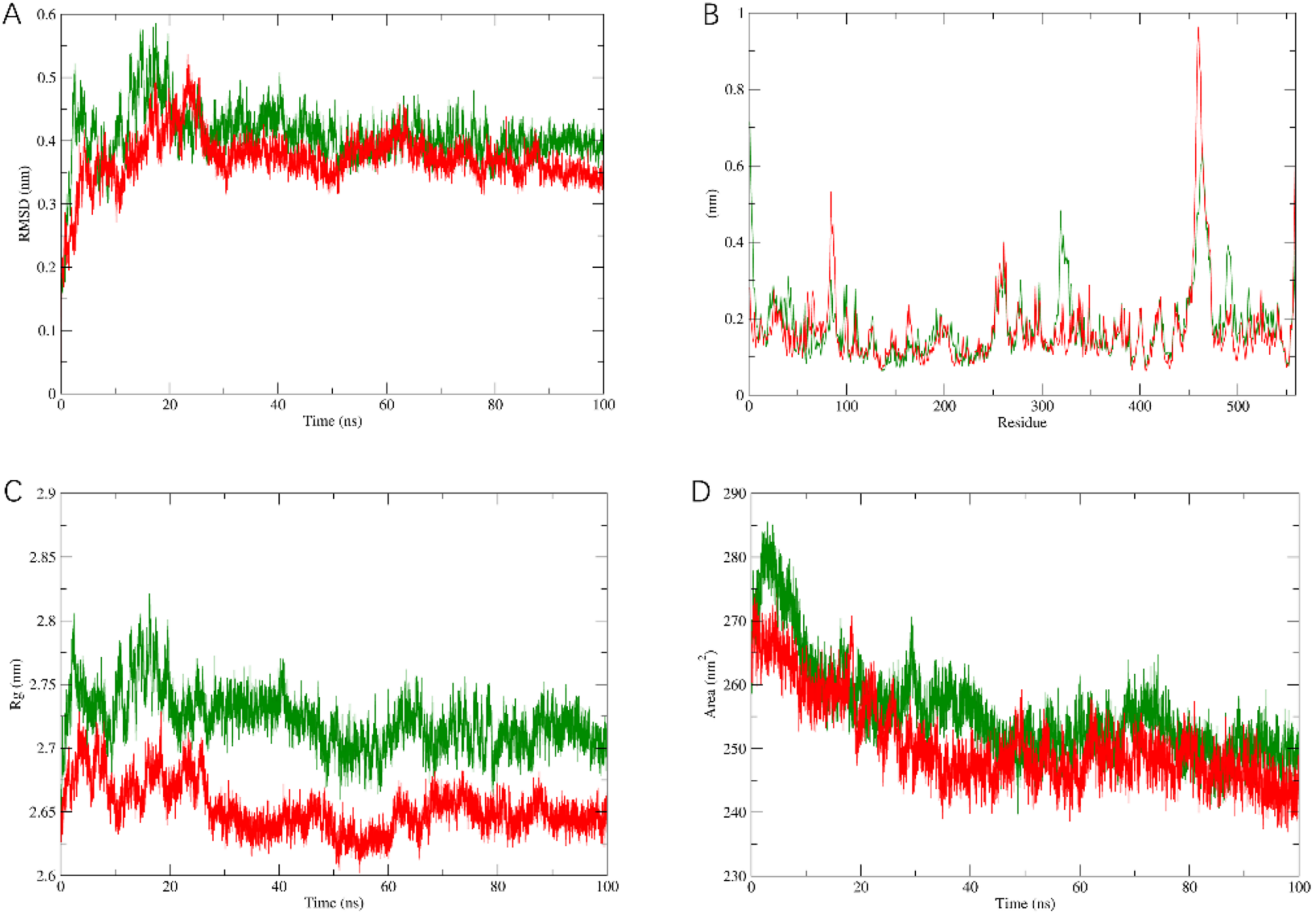
Molecular dynamics analysis of wild-type and mutant *TBC1D24* (wild-type: green, and mutant: red): (**A**) The trajectory of RMSD (root mean square deviation) of the two protein backbones. (**B**) RMSF (root mean square fluctuation) of each residue for the wild-type and mutant. (**C**) The radius of gyration (Rg) of the two proteins. (**D**) Variation and distribution of Solvent accessible surface area (SASA) for the proteins.

We calculated the root mean square deviation (RMSD) values of the wild-type and mutant protein backbones concerning the initial structure along the trajectory to assess the overall stability of the protein structure (Fig. 5A). Initially, both structures were highly similar, showing small RMSD values. The wild-type structure reached its maximum RMSD value at around 18 ns, while the mutant H487Y exhibited a peak at approximately 22 ns. The RMSD plot clearly indicates that the mutant is more stable than the wild-type.

Additionally, to compare the conformational flexibility of the wild-type and mutant proteins, we calculated the root mean square fluctuation (RMSF) values of the α-carbon atoms for each amino acid residue in both forms (Fig. 5B). The magnitude of RMSF reflects the flexibility of each residue during dynamics. The mutant displayed higher fluctuations in residues 450–500, whereas the wild-type residues exhibited relatively lower flexibility. Proper protein function relies on a specific stable structure, and mutation-induced changes in flexibility can impact functionality. This difference in stability may contribute to functional disparities between the mutant and wild-type.

We also examined the radius of gyration (Rg) plot (Fig. 5C) to demonstrate the effect of the mutation on protein compactness. A smaller Rg value indicates a more compact structure. We observed a significant decrease in the Rg value for the H487Y mutant, indicating an increase in protein compactness. Furthermore, the reduction in solvent accessibility surface area (SASA) suggests a decrease in structure size and surface area. Changes in SASA values reflect modifications in protein tertiary structure, with smaller SASA values indicating a denser protein structure. As depicted in Fig. 5D, the SASA curves of the wild-type and mutant proteins exhibited temporal variations. We observed that the SASA curve of the mutant protein was lower than that of the wild-type protein. This finding aligns with our expectations regarding the structural changes induced by the mutation.

### Regression analysis and statistical results

**Figure 6:**
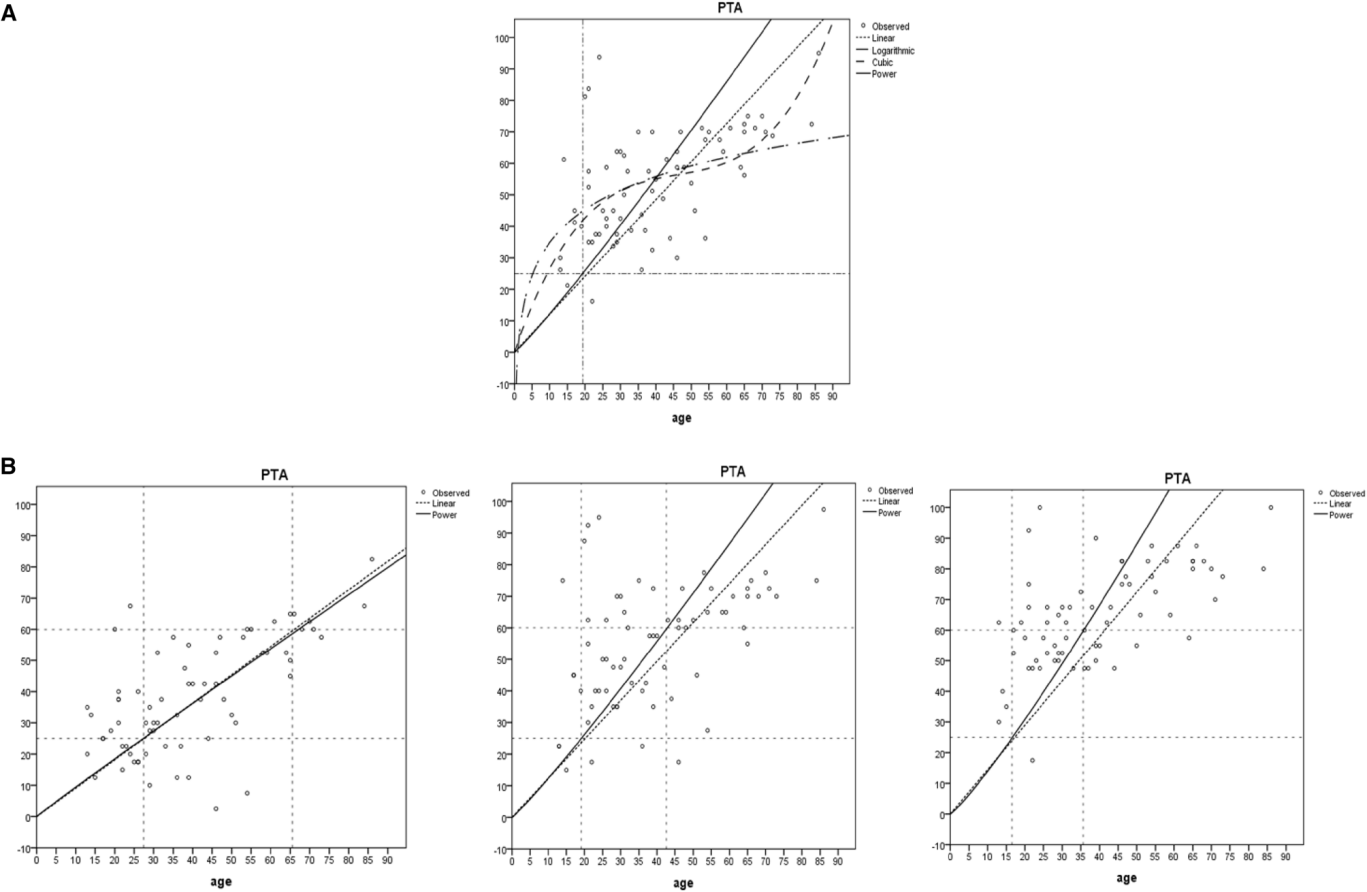
Phenotypic analysis. (**A**) Compares several different models: the power function model, the polynomial model, and the logarithmic model. The R-squared values of these models were 0.987, 0.933, and 0.930, respectively, while the linear regression model had an R-squared value of 0.873. The power function model had the best fit, so we chose it as our research model. (**B**) Shows the curve graphs of the power function fitting for the three frequency categories: low, mid, and high. we can obtain the years required for the hearing to deteriorate from 25 to 60 dB.

After a thorough investigation of the hearing-age correlation of *TBC1D24* hereditary non-syndromic hearing loss, we found that the power model provided the best fit. As shown in Fig. 6A, the model predicted the onset age as 19.23 years. By curve fitting with the power function model, we calculated the ages at which the hearing reached 25 dB and 60 dB for each frequency category. For low frequencies (250 Hz, 500 Hz), the ages were 26.07 years and 65.30 years. For mid frequencies (1000 Hz, 2000 Hz), the ages were 22.70 years and 41.17 years. For high frequencies (4000 Hz, 8000 Hz), the ages were 18.37 years and 37.74 years. These data enabled us to infer that the HPT for the low, mid, and high frequency categories were 39.23 years, 18.47 years, and 19.37 years, respectively, as shown in Fig. 6B.

In addition, we also found that there were significant differences in ATD between different domains at 2000 Hz and 4000 Hz frequencies (p < 0.05), as shown in Table 2. These findings provide important clues for understanding the pathogenesis of *TBC1D24* hereditary non-syndromic hearing loss.

**Table 2:** Comparison of ATDs by frequency between the two groups.

	0.25 kHz	0.5 kHz	1 kHz	2 kHz	4 kHz	8 kHz
TBC	0.7101	0.6897	0.6695	0.6778	0.7603	0.7781
TLDc	0.5690	0.3547	0.0581	0.0724	0.1016	0.4678
p-value	0.4476	0.1336	0.0150	0.0085	0.0004	0.1377

## Discussion

Congenital sensorineural hearing loss is typically caused by dysfunction in the inner ear. The normal development and function of the inner ear rely on the coordinated action of hundreds of genes that are specifically expressed in the inner ear. Variations in these inner ear genes can lead to genetic hearing loss with diverse clinical phenotypes and different mechanisms of deafness. The human *TBC1D24* gene (TBC1 domain family member 24, OMIM 613577) exhibits complex genetic pleiotropy, with various genotypic-phenotypic associations^21^. Different mutations can result in autosomal recessive nonsyndromic hearing loss (DFNB86)^11^ or autosomal dominant nonsyndromic hearing loss (DFNA65)^7,14^, early infantile epileptic encephalopathy 16 (EIEE16)^5^ with or without hearing loss, progressive myoclonic epilepsy (PME)^22^, familial infantile myoclonic epilepsy (FIME)^23^, and DOORS syndrome^24^. In this study, the proband III:2 presented severe bilateral sensorineural hearing loss (Fig. 1B) and carried a rare heterozygous missense mutation, c.1459C>T (p.His487Tyr) (Fig. 2B), in the *TBC1D24* gene. The mother II:1, who had a milder sensorineural hearing loss (Fig. 1C), inherited this mutation. The variant causes a substitution of histidine (H) with tyrosine (Y) at position 487, which is a highly conserved residue (Fig. 2A). While several mutations in the *TBC1D24* gene have been reported (Table 3), the involvement of *TBC1D24* in autosomal dominant inherited hearing loss seems to be relatively rare and specific. No mutations at this nucleotide position have been found in public databases. However, two mutations at the same amino acid position have been reported in a Polish family^25^, suggesting a possible hotspot region for mutations.

**Table 3:** Overview of genetic mutations in the *TBC1D24* gene.

Function class	Benign	Likely benign	Uncertain significance	Likely pathogenic	Pathogenic	Total
Missense	5	8	360	27	52	452
Nonsense	0	0	3	4	23	30
Frameshift	0	0	4	3	27	34
Inframe Indel	0	0	7	0	1	8
Splice junction loss	0	0	1	4	2	7

Table 3 provides a comprehensive overview of the genetic landscape of the *TBC1D24* gene through the analysis of known gene variants. These variants encompass a wide range of mutation types, including missense mutations, frameshift mutations, splice site mutations, and nonsense mutations. Each variant type has distinct effects on the protein structure and function, potentially leading to various clinical phenotypes.

*TBC1D24* is expressed in various tissues, including the inner ear, brain, kidney, heart, and liver, and its function may vary depending on the tissue and its interactions with different proteins. Rehman identified recessive mutations, c.208G>T (p.Asp70Tyr) or c.878G>C (p.Arg293Pro), in *TBC1D24* in four Pakistani families, resulting in non-syndromic deafness (DFNB86)^11^. Zhang discovered the p.S178L mutation in *TBC1D24* in a large pedigree with hereditary hearing loss^14^. The onset of hearing loss caused by this mutation occurred after the age of 20 and worsened progressively with age, particularly affecting high-frequency hearing. This differs from the clinical phenotype observed in this study. Although both cases involve dominant non-syndromic hearing loss, the proband in this family experienced onset around the age of 10, with more pronounced mid-frequency hearing impairment. This highlights the pleiotropy and complex genotype–phenotype correlation of the *TBC1D24* gene.

*TBC1D24*, the 24th member of the TBC1 domain family, was first discovered as a causative gene for epileptic seizures in an Italian family in 2010^23^. Mutations in the *TBC1D24* gene can affect the entire coding sequence and have been studied about inner ear expression in newborn and adult mice^7,14^. These studies suggest its crucial role in the development and maturation of hair cell stereocilia. In rats with silenced *TBC1D24* gene in neurons, disorders in axon growth and initial segment maturation were observed, impacting neuronal excitability^26^. *TBC1D24* is unique among the TBC/Rab-GAP family members as it contains the TLDc domain, which potentially regulates intracellular vesicle cycling and sorting^27^ while maintaining neuronal resistance to external stress^28^. Research indicates that mutations in the *TBC1D24* gene can impair neuronal endocytosis, affecting vesicle membrane trafficking and potentially leading to hearing loss^29^. Proteins with the TLDc domain play a protective role in enhancing cellular resistance to oxidative stress^28^. The identified variant in this study (p.His487Tyr) is located in the C-terminal region of the protein, corresponding to the TLDc domain. A frameshift variant involving this codon (p.His487Glnfs*71) has been reported in a patient with DOORS syndrome^30^. However, all known pathogenic variants of *TBC1D24* associated with autosomal dominant inherited deafness have been missense mutations so far.

The stability of the *TBC1D24* protein is influenced by various factors, including post-translational modifications, subcellular distribution, and interaction partners^28^. The *TBC1D24* gene consists of 8 exons, including an N-terminal TBC domain and a C-terminal TLD domain. The TBC domain functions as a GTPase-activating protein, catalyzing the hydrolysis of specific Rab-GTPases to GDP, thereby regulating vesicle membrane transport and sorting processes^31^. The TLD domain is also a highly conserved protein motif. Studies have found that proteins containing the TLD domain in mammals (*NCOA7, OXR1, TBC1D24, KIAA1609*) play a role in protecting cells against oxidative stress, highlighting the importance of the TLD domain in normal brain development and function. Interactions between its structural domains also affect the protein’s stability^28^. A study discovered weak interactions between the TBC and TLDc domains of *TBC1D24*, which can impact its stability^10^. Specifically, when these interactions are mutated or disrupted, the protein’s half-life decreases, leading to the formation of abnormal aggregates in cells. This suggests that the interactions between the TBC and TLDc domains play a role in the folding and function of *TBC1D24*^32^. Functional crosstalk exists between the TLDc and TBC domains, and mutations in the TLDc domain alone can affect the rest of the protein^27^. The conserved histidine residue at position 487, located near the long loop between the TBC and TLDc domains, may be involved in the binding between these domains, and its mutation could disrupt the formation of these complexes^25^. As observed from Fig. 4B, we can see that Tyr487 forms additional interactions with the residue Lys298, which is different from the wild-type residue His487. Lys298, as a binding site residue, is not only conserved but also plays a crucial role in the interaction between *TBC1D24* and other proteins such as IP3. It is deeply involved in these protein–protein interactions, highlighting its significance in the context of *TBC1D24*’s function^25^.

**Figure 7:**
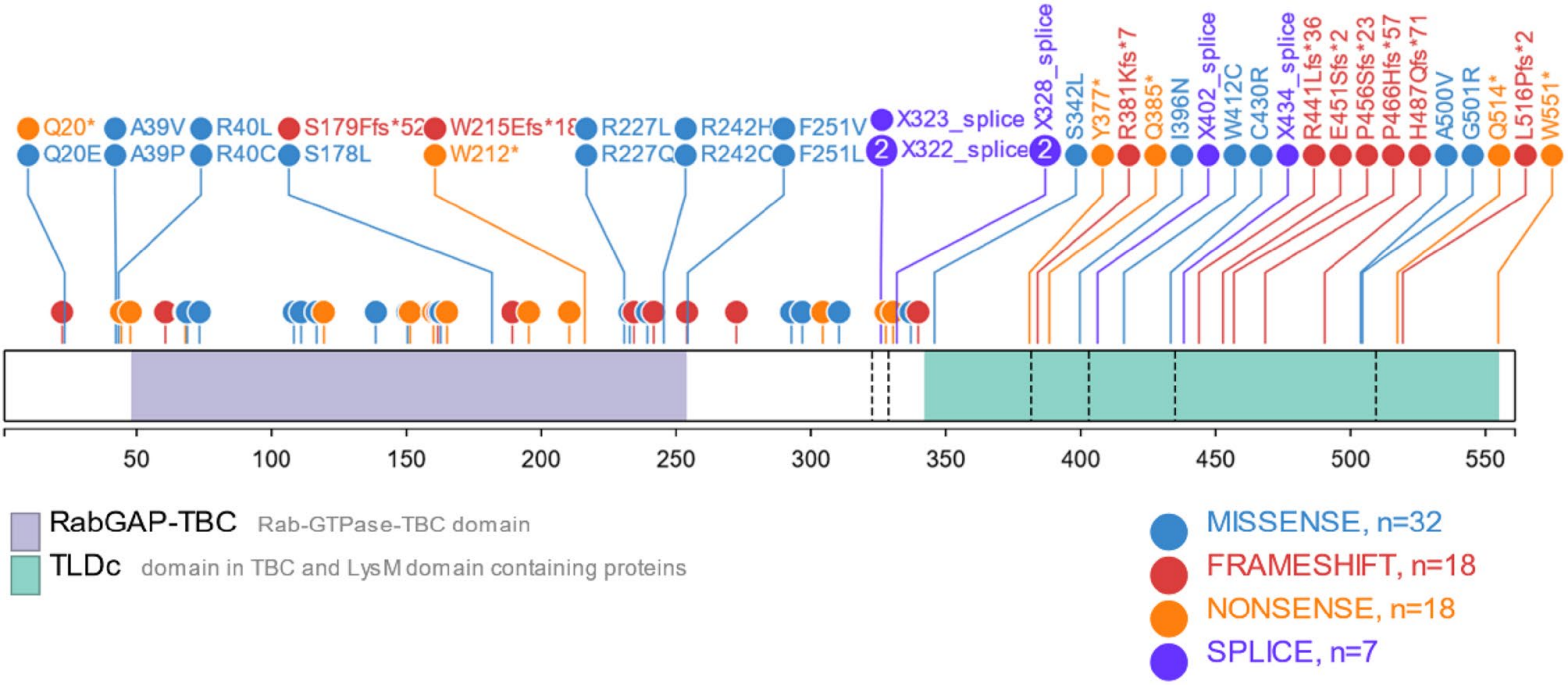
Mutations in the *TBC1D24* gene do not exhibit a clear genotype–phenotype correlation, making it difficult to predict the potential clinical phenotypes based on the location and type of mutations.

The *TBC1D24* gene exhibits widespread expression in tissues, and the uncertainty regarding the location of gene mutations leads to various clinical phenotypes, ranging from mild to severe. The pathogenesis of this gene is still unclear, and early diagnosis faces significant challenges. Pathogenicity of the *TBC1D24* gene can involve the entire coding sequence (Fig. 7), and no clear genotype–phenotype correlation has been observed^33^. Nonsense, frameshift, or splice site mutations can result in loss of protein function, leading to severe clinical phenotypes, while pathogenicity caused by missense mutations may be milder.

Research on patients with *TBC1D24* gene mutations has shown a correlation between the severity of axon formation defects and disease severity^26^. Literature reports describe two siblings with multifocal myoclonus and developmental delay, where compound heterozygous mutations were identified, including missense and frameshift mutations within *TBC1D24*^34^. The protein product of the *TBC1D24* gene acts as an ADP-ribosylation factor 6 (ARF6) binding partner, exerting its function by regulating the activity of ARF6. ARF6 plays a crucial role in dendritic branching, spinal formation, and axon extension in the nervous system^35^. Pathogenic mutations in the *TBC1D24* gene disrupt its binding with ARF6, leading to severe impairment of neuronal development. Studies on *TBC1D24* mutant mice have revealed that although overall neural development is normal, there are significant effects on neuronal maturation and antioxidant stress response, indicating that mutations in conserved protein domains of *TBC1D24* may interfere with neuronal development^29^. According to dynamic simulation results, the 487th amino acid of the *TBC1D24* protein may play an important role in maintaining its stability. Proteins require a specific stable structure to perform their biological functions, and this mutation alters its stability, affecting its binding or interaction with downstream proteins. In African clawed frog research, *TBC1D24* indirectly interacts with EphrinB2 through the Dishevelled protein, influencing cranial neural crest migration^36^. Different types of *TBC1D24* gene mutations can cause complete or partial loss of protein function or harmful changes in specific protein functions, resulting in functional impairments in different tissues and systems through the disruption of various biological pathways^37^. Heterozygous carriers of truncating *TBC1D24* gene mutations in a certain family exhibit a normal clinical phenotype, suggesting that the *TBC1D24* gene mutation p.Ser178Leu associated with non-syndromic dominant deafness is likely a gain-of-function mutation^38^. This mutation alters the folding or structure of the protein, resulting in detrimental changes to its function. By analyzing and comparing protein stability, flexibility, and compactness, and employing molecular dynamics to study conformational changes in mutant proteins, we can gain insights into the pathogenic mechanisms of mutations and aid in drug design at a more microscopic level. Epilepsy is the most common clinical symptom associated with *TBC1D24* gene mutations, but most patients have a poor response to anti-epileptic drugs^39^. Tona designed a mouse model with a premature termination codon similar to humans, which can be used to screen anti-epileptic drugs for their effectiveness against seizures associated with *TBC1D24* gene mutations^40^.

Non-syndromic hearing loss caused by *TBCD124* gene mutation shows variable onset age. The self-reported onset age of hearing loss is prone to be influenced by factors such as recall bias^41^. The symptoms of the disease are subtle in the early stage, and mild hearing loss often does not attract the attention of the patients, leading to delayed medical treatment. The onset age calculated by non-linear regression analysis reflects the actual average onset age more reliably than the self-reported average onset age. This calculated onset age may help to determine the optimal treatment time.

In previous studies of other types of hereditary hearing impairment, we found that the hearing loss progression speed differed among different frequencies^42^. Compared with low frequencies, the hearing impairment of affected individuals progressed faster in mid and high frequencies. We conducted a thorough investigation of patients with non-syndromic deafness caused by *TBC1D24* gene mutation and compared the HPT of low, mid, and high frequencies. The results showed that the HPT of low frequencies was significantly longer, while that of mid and high frequencies was shorter. These findings may help clinicians to prescribe treatment plans to delay or stop the progression of hearing loss. The genotype–phenotype relationship of *TBC1D24* patients is not clear, and similar mutations may lead to various phenotypic differences, which is also common in other gene mutation studies^43^. Because non-syndromic hearing loss has no obvious external phenotype, such as physical defects, it is difficult to infer the mutation location in the genotype by phenotype. We calculated the pure tone hearing threshold data of TBC and TLDc domains and innovatively found that ATD also differed at 2000 Hz and 4000 Hz, two higher frequencies. However, puzzlingly, the ATD difference between the two domains was not significant at 8000 Hz frequency. This provides some clues to investigate the pathogenic mechanism of hearing loss associated with mutations in the *TBC1D24* gene, but more detailed clinical data are needed to validate this result.

In conclusion, we report a rare heterozygous mutation in the *TBC1D24* gene found in a Chinese family affected by autosomal dominant non-syndromic hearing loss. This discovery adds to the growing range of pathogenic genes associated with *TBC1D24*. Furthermore, we have conducted preliminary investigations into the molecular mechanisms underlying the mutation and its effect on hearing loss. However, given the extensive variability and apparent phenotypic heterogeneity of *TBC1D24*, further research is needed for our future endeavors.

## Data Availability

The patient’s phenotype and the detected variant data have been submitted to ClinVar (https://www.ncbi.nlm.nih.gov/clinvar/), and the Submission ID is SUB12678122.
